# Assessing the Necessity and Cost-effectiveness of Radiology Reports for Plain Hand Radiographs in Outpatient Clinics

**DOI:** 10.1177/22925503241301722

**Published:** 2024-12-10

**Authors:** Bader Al-Zeer, Ahmed Al Hosni, David Tang

**Affiliations:** 1 Faculty of Medicine, The University of British Columbia, Vancouver, BC, Canada; 2 Division of Plastic Surgery, 8167 Vancouver General Hospital, Vancouver, BC, Canada

**Keywords:** cost-effectiveness, hand clinic, hand radiographs, plastic surgery, quality improvement, rentabilité, clinique de la main, radiographies des mains, chirurgie plastique, amélioration de la qualité.

## Abstract

**Purpose:** The purpose of this study was to determine the necessity and cost-effectiveness of radiologists’ interpretation of plain hand radiographs for diagnosing and managing different hand pathologies in the plastic surgery outpatient clinic setting. **Methods:** Through a retrospective cohort study, we identified new patient encounters from January 2021 to December 2022 in an outpatient hand clinic. We included patients with radiology reports that were submitted subsequent to the surgeon's consult note in clinic. Plastic surgeons’ interpretations were compared with radiology reports, and findings were categorized as concordant, discrepant (different with no impact on management), or discordant (different with an impact on management). Analysis included radiograph type, underlying mechanism, site of pathology, and hand fellowship training for surgeons. Direct cost was calculated using billing code fees. **Results:** A total of 241 cases were included in the study: 187 were concordant (77.6%), 53 were discrepant (22%) and only one was discordant (0.4%). Trauma was the predominant reported mechanism (90.5%). Most pathologies were in the phalanges (53.5%), followed by metacarpals (30.7%) and carpals (15.8%). Univariate analysis demonstrated no significant association between radiograph type (*P* = .829), underlying mechanism (*P* = .172), site of pathology (*P* = .482), surgeon's training (*P* = .551) and concordance with radiology reports. Total direct cost of radiographs reporting in this study was 8477 CAD. **Conclusion:** The study identified high concordance rates of hand radiograph findings between plastic surgeons and radiologists, with radiology reports rarely impacting patient management. Given the significant patient volume in plastic surgery hand clinics, these findings prompt further considerations in optimizing cost-effective diagnostic practices.

## Résumé

**Objectif :** L’objectif de cette étude était de déterminer le besoin et la rentabilité de l’interprétation par un radiologiste des radiographies standard de la main pour le diagnostic et la gestion des différentes pathologies de la main dans un cadre de consultations externes de chirurgie plastique. **Méthodes :** Nous avons identifié pour une étude rétrospective de cohorte de nouveaux patients vus de janvier 2021 à décembre 2022 dans une consultation externe de la main. Nous avons inclus des patients avec des rapports de radiologie qui étaient soumis après une note de consultation du chirurgien de la clinique. Les interprétations des chirurgiens plasticiens ont été comparées aux rapports de radiologie et les constatations ont été classées en « concordant », « divergent » (différent mais sans répercussion sur la gestion), ou « discordant » (différent avec répercussion sur la gestion). L’analyse a inclus le type de radiographie, le mécanisme sous-jacent, le site de la lésion et la formation de fellowship de la main pour chirurgiens. Le coût direct a été calculé en utilisant les montants liés aux codes de facturation. **Résultats :** Un total de 241 cas ont été inclus dans l’étude : 187 cas (77,6%) étaient concordants, 53 cas (22%) étaient divergents et seulement un cas (0,4%) était discordant. Un traumatisme était le mécanisme signalé prépondérant (90,5%). La plupart des atteintes concernaient les phalanges (53,3%) suivies des métacarpes (30,7%) et des os du carpe (15,8%). Une analyse unifactorielle a montré qu’il n’y avait pas d’association significative entre le type de radiographie (*P *= 0829), le mécanisme sous-jacent (*P *= 0172), le site de la lésion (*P *= 0482), la formation du chirurgien (*P *= 0551) et la concordance avec les rapports de la radiologie. Le coût direct total des rapports de radiographies dans cette étude a été de 8 477 CAD. **Conclusion :** L’étude a identifié des taux de concordance élevés des constatations radiographiques de la main entre les chirurgiens plasticiens et les radiologistes, avec des rapports de radiologie ayant rarement des répercussions sur la gestion des patients. Compte tenu du volume significatif de patients dans les cliniques de chirurgie plastique de la main, ces constatations soulèvent la question d’une optimisation de la rentabilité des pratiques diagnostiques.

## Introduction

With the continuously increasing costs in healthcare, balancing cost-effectiveness while maintaining quality of care is a critical and ongoing objective in medicine. Whether within a universal healthcare system like Canada's or the private systems seen globally, the availability of funding and physicians is limited and must be used efficiently. Therefore, regular evaluation of clinical and administrative practices is crucial to maintaining balance within the ever-changing landscape of healthcare needs and resources.

Radiology reports are essential for communication between radiologists and specialists, particularly for complex patients and advanced imaging modalities. However, in areas of shared expertise, such as plain radiograph interpretation, duplication of efforts between surgeons and radiologists warrants evaluation. Typically, radiologists generate a report interpreting all radiographs, but whether surgeons review these reports has been the subject of several studies.^1–3^ Additionally, the utility of these reports has come under scrutiny in multiple orthopedic studies, which highlight issues such as delayed report availability, incorrect or missed findings, limited impact on patient care, and a lack of new clinically relevant information compared to the surgeon's note.^4–10^

Hand trauma occurs with considerable frequency and is associated with significant morbidity and financial burden.^11–13^ Plain radiographs are the first line of investigation for most of these injuries. At our center, a high-volume, fast-paced hand clinic operates weekly, where most patients undergo hand radiography shortly before being seen by a plastic surgeon who then decides the management plan. While previous studies have assessed the utility of radiology reports for lower extremity trauma, there is a scarcity of literature exploring this issue in hand trauma. This study aims to determine the necessity and cost-effectiveness of radiologists’ interpretation of plain hand radiographs in the management of various hand pathologies by plastic surgeons and plastic surgery trainees in a high-volume academic center setting.

We hypothesize that the interpretation of plain hand radiographs by plastic surgeons and plastic surgery trainees does not differ significantly from that of radiologists. Additionally, we hypothesize that radiology reports rarely result in changes to patient management in outpatient hand clinics.

## Methods

Following ethics approval from the UBC Research Ethics Board (ID: H23-00228), we conducted a retrospective cohort chart review of all patients seen at the outpatient hand clinic at our tertiary care center from January 2021 to December 2022.

The hand clinic schedule was used as the primary source to identify eligible patients. Inclusion criteria were: (a) adult patients (>18 years) seen at the tertiary care center's hand clinic between January 2021 and December 2022, (b) patients being seen for a new presentation with first-time radiographs ordered in the clinic, and (c) the timestamp of the surgeon signing the clinic note was before the timestamp of the radiologist signing the radiology report. Exclusion criteria were: (a) patients who had any hand radiograph with a radiology report available before the surgeon signed the clinic note, and (b) evidence of communication between the radiologist and hand surgeon.

We collected the following data: clinic visit date, timestamp of clinic note signed by the surgeon, whether the surgeon had hand fellowship training, clinic diagnosis, anatomical location (carpals, metacarpals, phalanges), underlying mechanism (trauma, degenerative, infectious), management plan, type of radiograph (finger, hand, wrist), timestamp of the radiology report signed by the radiologist, and diagnosis in the radiology report.

Plastic surgeons’ interpretations were compared with radiology reports, and findings were categorized as follows: (a) concordant: both surgeon and radiologist identified the same findings, (b) discrepant: differences in the level of relevant information reported or interpretation without impact on clinical management, and (c) discordant: differences in the level of relevant information reported or interpretation with impact on clinical management. Management plans were reviewed at follow-up visits for all discrepant and discordant cases.

We used the Chi-squared test to examine the association between radiograph type, underlying mechanism, site of pathology, hand fellowship training for surgeons, and concordance with radiology reports. Analyses were performed using R Studio Statistical Software (v.4.2.2; R Core Team 2022). The direct cost of radiology reports was calculated using British Columbia's provincial billing code fee.

## Results

Over the 2-year study period, there were 241 instances where a new patient was evaluated and treatment was initiated by the plastic surgery team at the hand clinic before the radiology report for the radiograph was signed. The median time between the submission of the clinic note by the surgeon and the radiology report was 49 minutes (IQR: 22-111 minutes). Of the 241 cases, 187 (77.6%) were concordant, 53 (22%) were discrepant, and only one case (0.4%) was discordant.

Available follow-up clinic visit notes and follow-up radiography reports were reviewed for all discrepant and discordant cases. Of the 53 discrepant cases: 13 had additional findings in the radiology report related to the presentation, 19 had additional findings in the radiology report unrelated to the presentation, 8 had additional findings in the surgeon's note related to the presentation, and 13 had different findings. Discrepancies in these cases did not warrant a change to the surgeon's initial management plan. In the single discordant case, the patient presented with tenderness over the snuffbox following a fall on their hand on ice. Given the mechanism of injury and clinical exam, the surgeon suspected a possible scaphoid fracture, which was not noted in the radiology report. However, due to the uncertainty, it was the surgeon who ordered further imaging and decided to remove the wrist splint at a follow-up visit.

The majority of cases involved the phalanges (53.5%), followed by the metacarpals (30.7%) and carpals (15.8%). The underlying mechanisms were trauma, degenerative conditions, and infections, with trauma being the most commonly reported mechanism (90.5%). There were 9 plastic surgeons included in this study and most cases (80.5%) were seen by a plastic surgeon with hand fellowship training. Hand radiographs were performed in about half of the cases (54.8%), while finger and wrist radiographs were done in 32.4% and 12.9% of the cases, respectively.

No pattern was identified for concordance distribution within each subset of variables. Statistical analysis demonstrated no significant association between radiograph type (*P* = .829), underlying mechanism (*P* = .172), site of pathology (*P* = .482), surgeon's training (*P* = .551), and concordance with radiology reports.

The cost per radiology report in British Columbia's billing system is 35.32 CAD. Consequently, the total cost for the 241 reports identified in this study amounts to 8477 CAD. Based on our clinic's schedule, we estimate approximately 1860 new cases per year. Therefore, the annual cost for the radiology report of the first radiograph for all these cases would be 69,695 CAD at our center.

## Discussion

This study assessed the necessity and cost-effectiveness of radiologists’ interpretations of plain hand radiographs in a plastic surgery outpatient clinic. We found a high concordance rate between surgeons’ clinic notes and radiology reports, with radiology reports impacting patient management in only 0.4% of cases.

Similar to our findings, delays in the availability of plain radiograph reports have been reported in the literature.^2,8^ Du et al observed delays in 32% of cases in their study evaluating the value of radiograph reports in an outpatient clinic setting.^8^ Donners et al also highlighted this issue, reporting that 24% of orthopedic surgeons cited delayed report availability as a reason for not consulting radiograph reports.^2^ Additionally, 19% of surgeons in the same study indicated a general lack of time as a barrier to utilizing these reports.^2^ In our cohort, the delays could be attributed to the fast-paced nature of our clinic and the surgeons’ confidence in initiating treatment plans based solely on radiographs without waiting for the report. As with other subspecialties, the constant high-volume exposure to radiographs, combined with the ability to clinically examine patients, allows plastic surgeons to confidently interpret hand radiographs independent of the radiology report.

The high concordance rate and low incidence of management changes due to differences between surgeons’ and radiologists’ interpretations of radiographs in our cohort align with findings from previous studies.^8,9,14–16^ Du et al compared radiologist and surgeon interpretations of radiographs for 441 pediatric supracondylar fracture patients in an outpatient clinic and found that radiologist interpretations did not change orthopedic management in any case.^8^ Similarly, Nayak et al conducted a prospective study of 516 total knee or hip replacement procedures and identified only two cases where management was altered based on pathologies identified by the surgeon in post-operative radiographs.^14^ Catapano et al reviewed 13,561 cases of minor extremity trauma evaluated by an attending orthopedist in the absence of a radiologist during weekends or overnight and reported that only 1.1% of cases required a change in management following radiologist interpretation.^15^ Turn et al also found no significant difference in interpretations in their interdisciplinary prospective study, where an orthopedic surgeon and a radiologist independently reviewed 507 musculoskeletal radiographs without conducting a physical exam.^16^ Collectively, these studies suggest that radiology reports of musculoskeletal radiographs interpreted by surgeons have minimal impact on patient management.

Our findings support the recommendations of previous studies that dual-interpretation of musculoskeletal plain radiographs is neither necessary nor cost-effective, especially in high-volume, low-acuity outpatient clinics like ours.^7–9,14^ The annual cost of hand radiograph reports estimated in our study (65,695 CAD) does not account for the cost of follow-up radiographs that many patients would require, which would further increase the total cost per patient. Given the limited funding and availability of radiologists, these resources could be better utilized for more complex presentations and imaging modalities. Further, radiology reports for hand radiographs in outpatient clinics could become an option that the treating surgeon can request as needed.

To our knowledge, this is the first study to explore the necessity of dual-interpretation of plain radiographs in hand pathologies seen in outpatient clinics. Previous studies on this issue have focused solely on orthopedic surgeons and not plastic surgeons. Nevertheless, this study has multiple limitations, including its retrospective design and only including adult patients. By only including cases with no radiology report available before the surgeon's clinic note, this study attempted to control for the impact of radiologists on initial management decisions. However, we acknowledge the possibility of undocumented direct communication between the surgeon and radiologist, although this is likely rare. Lastly, it's important to note that this study focused on a high-volume center where most patients were seen by hand fellowship-trained plastic surgeons, which may limit the generalizability of the findings.

## Conclusion

**Figure 1.**
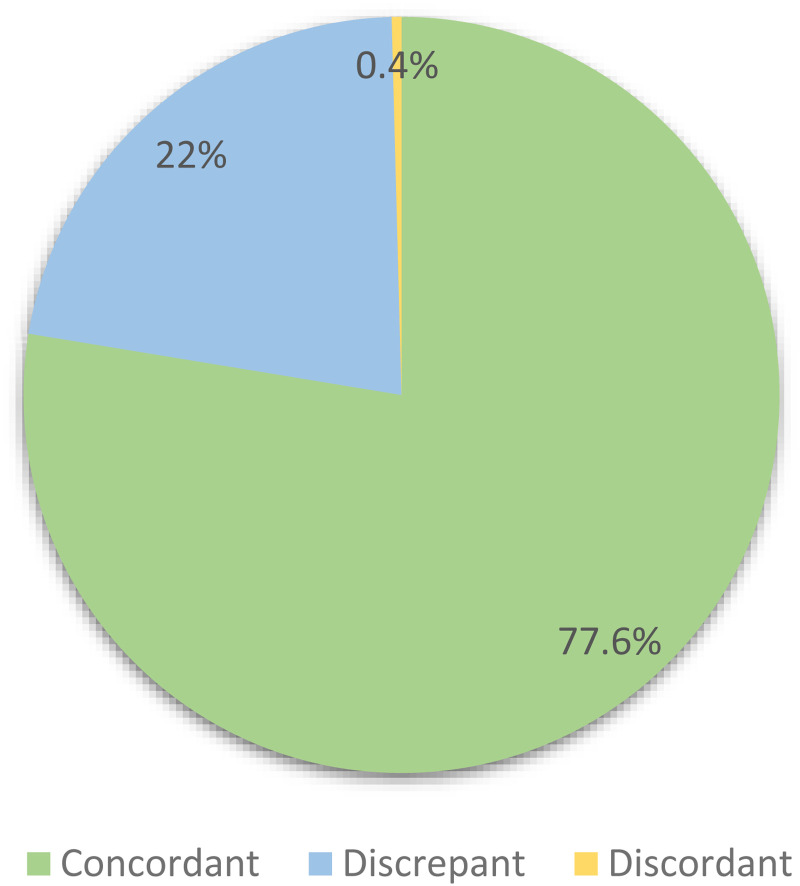
Distribution of concordance categories between plastic surgeon's clinic notes and subsequent radiology reports for new patients seen at the hand clinic at our tertiary care center between January 2021 and December 2022 (*n* = 241).

**Figure 2.**
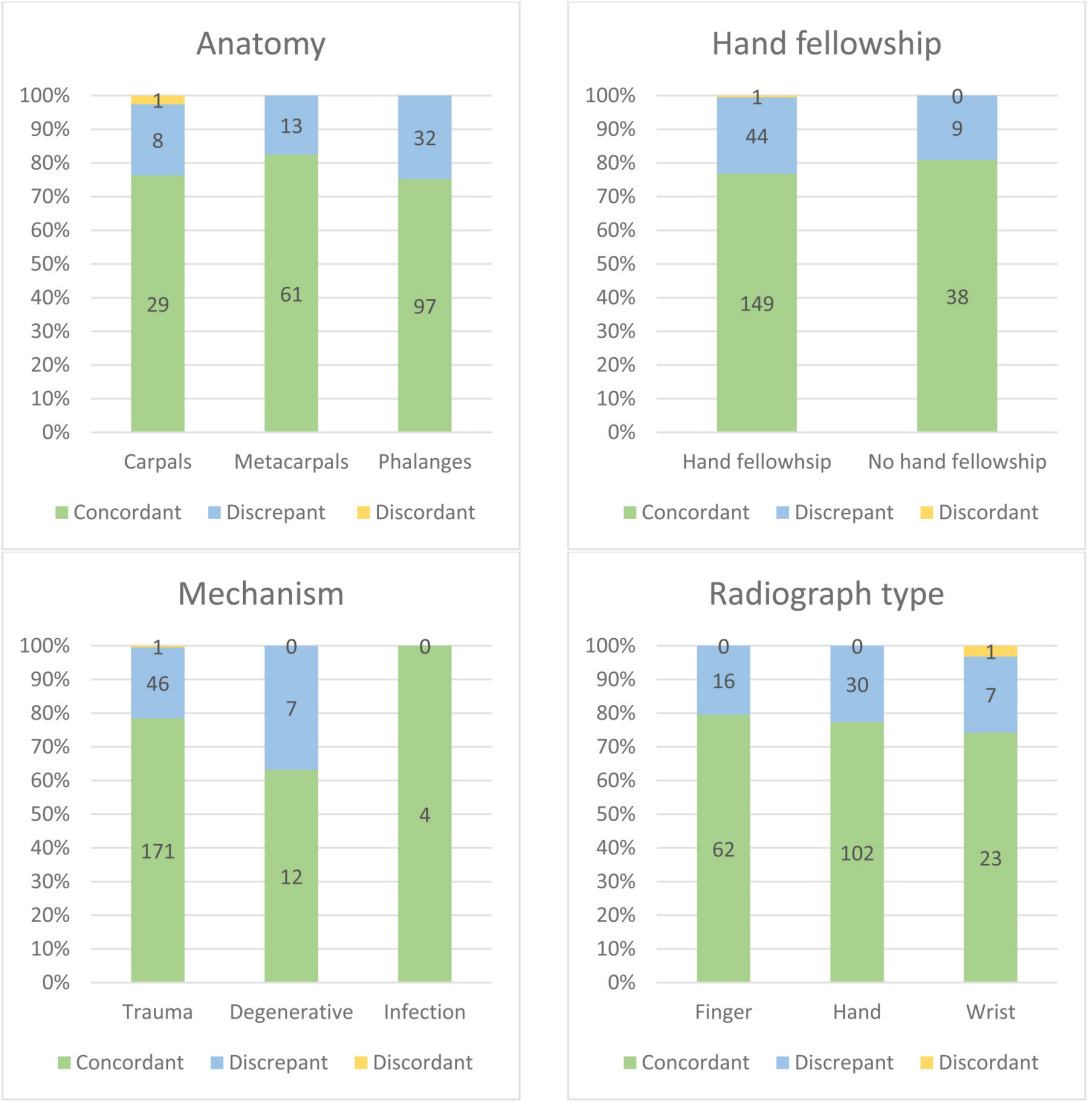
Distribution of new patients seen at the hand clinic at our tertiary care center between January 2021 and December 2022 with radiology reports signed after the respective clinic notes (*n* = 241). Cases are categorized by anatomical site, surgeon's hand fellowship training, underlying mechanism, and radiograph type. The charts show concordance categories (concordant, discrepant, discordant) as total numbers within each subset of variables.

This study examined hand cases diagnosed and treated by plastic surgeons in an outpatient clinic before radiograph reports were available, revealing a high concordance rate with radiology reports and negligible impact on patient management. This suggests that plastic surgeons can effectively treat hand pathologies without relying on radiology reports. Our cost analysis highlights significant annual expenditures on radiology reports, emphasizing the need for cost-effective diagnostic practices. Despite its retrospective design and focus on a high-volume center, this study adds to the limited literature on this issue by focusing on hand radiograph interpretation by plastic surgeons. Future research should include the perspectives of plastic surgeons and surgical trainees on their use of radiology reports for hand radiographs. Factors such as type and location of practice, fellowship training, hand cases volume, and years of practice or training should be considered. Increasing the sample size will also be important to identify potential areas of discordance and refine recommendations accordingly. Additionally, calculating the total cost of all hand radiograph reports and the corresponding radiologists’ time is essential to better inform resource allocation. Finally, the medicolegal implications must be explored before implementing changes to current practices (Figures 1 and 2).
